# Antidepressant Effects of Aripiprazole Augmentation for Cilostazol-Treated Mice Exposed to Chronic Mild Stress after Ischemic Stroke

**DOI:** 10.3390/ijms18020355

**Published:** 2017-02-08

**Authors:** Yu Ri Kim, Ha Neui Kim, Ki Whan Hong, Hwa Kyoung Shin, Byung Tae Choi

**Affiliations:** 1 Department of Korean Medical Science, School of Korean Medicine, Pusan National University, Yangsan 50612, Korea; sprout_ing@pusan.ac.kr (Y.R.K.); Kim.Haneui@mayo.edu (H.N.K.); 2 Korean Medical Science Research Center for Healthy-Aging, Pusan National University, Yangsan 50612, Korea; 3 Department of Pharmacology, School of Medicine, Pusan National University, Yangsan 50612, Korea; kwhong@pusan.ac.kr; 4 Division of Meridian and Structural Medicine, School of Korean Medicine, Pusan National University, Yangsan 50612, Korea

**Keywords:** aripiprazole, cilostazol, depression, stroke, chronic mild stress

## Abstract

The aim of this study was to determine the effects and underlying mechanism of aripiprazole (APZ) augmentation for cilostazol (CLS)-treated post-ischemic stroke mice that were exposed to chronic mild stress (CMS). Compared to treatment with either APZ or CLS alone, the combined treatment resulted in a greater reduction in depressive behaviors, including anhedonia, despair-like behaviors, and memory impairments. This treatment also significantly reduced atrophic changes in the striatum, cortex, and midbrain of CMS-treated ischemic mice, and inhibited neuronal cell apoptosis, particularly in the striatum and the dentate gyrus of the hippocampus. Greater proliferation of neuronal progenitor cells was also observed in the ipsilateral striatum of the mice receiving combined treatment compared to mice receiving either drug alone. Phosphorylation of the cyclic adenosine monophosphate response element binding protein (CREB) was increased in the striatum, hippocampus, and midbrain of mice receiving combined treatment compared to treatment with either drug alone, particularly in the neurons of the striatum and hippocampus, and dopaminergic neurons of the midbrain. Our results suggest that APZ may augment the antidepressant effects of CLS via co-regulation of the CREB signaling pathway, resulting in the synergistic enhancement of their neuroprotective effects.

## 1. Introduction

Depression is a debilitating mental disorder characterized by negative mood and diminished interest or pleasure in daily activities [1]. The hypothesis of vascular depression proposes an association between cerebrovascular diseases such as stroke and an increased risk for depression due to injuries of the brain [2,3,4,5]. Depression after a stroke is a subtype within the broad category of vascular depression, and was found to share common underlying mechanisms with other nonvascular types of depression in rats [6]. More than one-third of stroke survivors show mood symptoms, and post-stroke depression is clinically important because it is associated with an increase in mortality and decrease in functional recovery [1,7].

The monoamine hypothesis of depression focuses on changes in the levels of serotonin, norepinephrine, dopamine, and cortisol as being of central importance to the pathophysiology of depression [8,9]. However, current biological theories of post-stroke depression posit that even small focal vascular damage, such as that caused by ischemic brain injury, can contribute to the clinical symptomatology of depression. Disruption of the fiber tracts connecting the prefrontal cortex, striatum, nucleus accumbens, hippocampus, thalamus, and amygdala is involved in the regulation of mood and cognition of post-stroke depression [1,10,11].

Aripiprazole (APZ) is a new atypical antipsychotic drug with a unique pharmacological profile, acting as a partial serotonin and dopamine agonist [12,13]. APZ is employed usually as an adjunct therapy in combination with a drug from the group of selective serotonin reuptake inhibitors in the treatment of major depressive disorders [13,14]. Cilostazol (CLS) possesses a powerful means to produce various pleiotropic effects through the elevation of intracellular cyclic adenosine monophosphate (cAMP) levels, resulting in the increased phosphorylation of the cAMP response element binding protein (CREB) [15,16,17]. CLS has been widely approved for the secondary prevention of ischemic stroke [18].

Long-term stressful conditions after a stroke enhance tissue injury and contribute to the severe clinical symptoms of depression [19,20,21]. Thus, prevention of secondary neurodegeneration may be one of the clinical benefits of antidepressant therapy after a stroke in regards to both functional recovery and survival [1,22]. CLS provides protective effects during ischemic injury through the activation of anti-apoptotic signaling pathways [23,24]. Psychopharmacotherapy with antidepressants provides an effective treatment for depression by control of the selective activation of monoamine receptors, but mood stabilizers involving APZ may also exert antidepressant effects by promoting brain neuroprotection and neurogenesis [25].

Thus, although APZ and CLS have completely different signaling pathways that underlie their formal efficacy, both may exert antidepressant activities at least in part via effects on shared pathways controlling neuronal survival or neurogenesis. Therefore, we hypothesized that APZ may show synergistic antidepressant actions when combined with the neuroprotective agent CLS. To validate this hypothesis, we focused on the effects of active doses of APZ administered in combined treatment with a sub-active dose of CLS. To examine the effects of these drugs on stress-induced depression following ischemic stroke, we have investigated the effects of this combined treatment on chronic mild stress (CMS)-treated mice after middle cerebral artery occlusion (MCAO).

## 2. Results

### 2.1. Treatment Effects on the Depressive Behavioral Phenotypes

**Figure 1:**
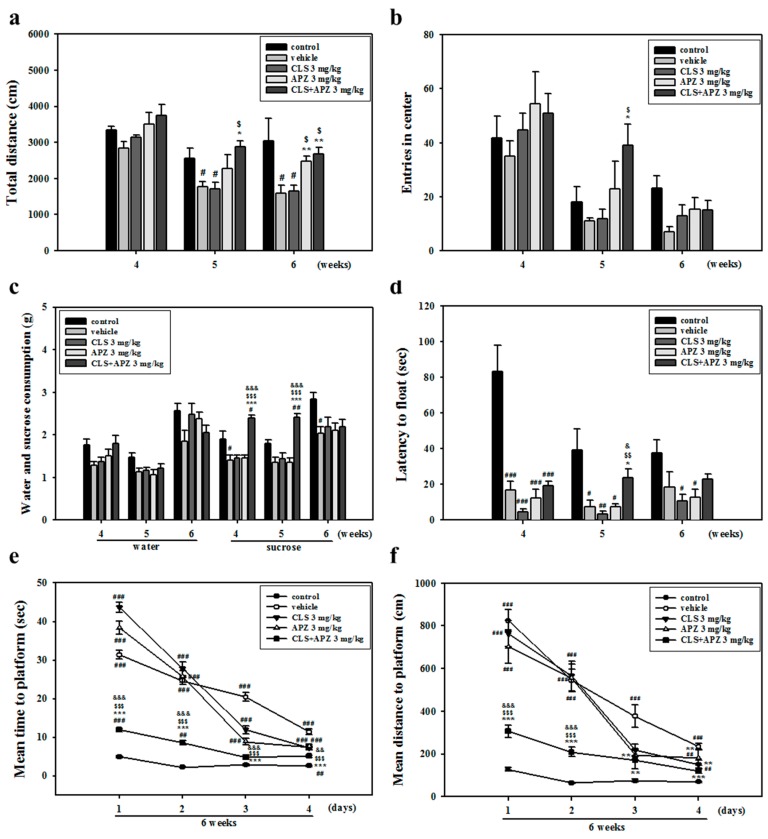
Effect of aripiprazole (APZ) augmentation on depressive behaviors in cilostazol (CLS)-treated mice. Open field (**a**,**b**) (*n* = 6), sucrose preference (**c**) (*n* = 5), forced swim (**d**) (*n* = 5) and Morris water maze tests (**e**,**f**) (*n* = 6) were performed in CMS-treated mice after ischemic stroke. Combined treatment resulted in significant recovery from depressive behaviors such as anhedonia, despair and impairment of spatial memory. Data are presented as mean ± standard error (SEM). ^#^ *p* < 0.05, ^##^ *p* < 0.01 and ^###^ *p* < 0.001 versus control mice; * *p* < 0.05, ** *p* < 0.01 and *** *p* < 0.001 versus vehicle-treated mice; ^$^ *p* < 0.05, ^$$^ *p* < 0.01 and ^$$$^ *p* < 0.001 versus CLS-treated mice; ^&^ *p* < 0.05, ^&&^ *p* < 0.01 and ^&&&^ *p* < 0.001 versus APZ-treated mice.

In the open field test, total distance decreased significantly in vehicle-treated mice, compared to the control mice. However, locomotor activity was higher after combined treatment, compared to treatment with vehicle or CLS alone (Figure 1a: 5 weeks, F_(4,23)_ = 5.607, *p* = 0.003; 6 weeks, F_(4,20)_ = 4.030, *p* = 0.022). CMS-treated MCAO mice treated with APZ and CLS showed significantly higher center entry numbers compared to mice treated with either vehicle or CLS alone (Figure 1b: 5 weeks, F_(4,25)_ = 3.177, *p* < 0.031). In vehicle-treated mice, sucrose consumption in the sucrose preference test and latency to float in the forced swim test were also significantly decreased, compared to the control group. Mice treated with APZ and CLS showed significantly higher sucrose intake and time floating compared to mice treated with the vehicle, APZ alone, or CLS alone (Figure 1c: 4 weeks, F_(4,20)_ = 15.399, *p* < 0.001; 5 weeks, F_(4,20)_ = 17.271, *p* < 0.001, Figure 1d: 5 weeks, F_(4,20)_ = 6.408, *p* = 0.002). In the Morris water maze test, vehicle-treated mice took a longer time and swam a greater distance on average to find the platform compared to control mice, but mice treated with APZ and CLS attained a significantly lower escape latency compared to vehicle and APZ- or CLS-treated mice (Figure 1e: 1 day, F_(4,25)_ = 243.532, *p* < 0.001; 2 days, F_(4,25)_ = 107.698, *p* < 0.001; 3 days, F_(4,25)_ = 73.570, *p* < 0.001; 4 days, F_(4,25)_ = 39.956, *p* < 0.001; Figure 1f: 1 day, F_(4,25)_ = 33.154, *p* < 0.001; 2 days, F_(4,25)_ = 24.379, *p* < 0.001; 3 days, F_(4,25)_ = 10.071, *p* < 0.001; 4 days, F_(4,25)_ = 23.088, *p* < 0.001). These results suggest that compared to treatment with APZ or CLS alone, the augmentation of APZ with CLS showed a marked reversal of depressive behavioral phenotypes, including anhedonia, despair-like behaviors, and memory impairments, but not anxiety-like behaviors.

### 2.2. Treatment Effects on the Atrophic Changes of the Brain

**Figure 2:**
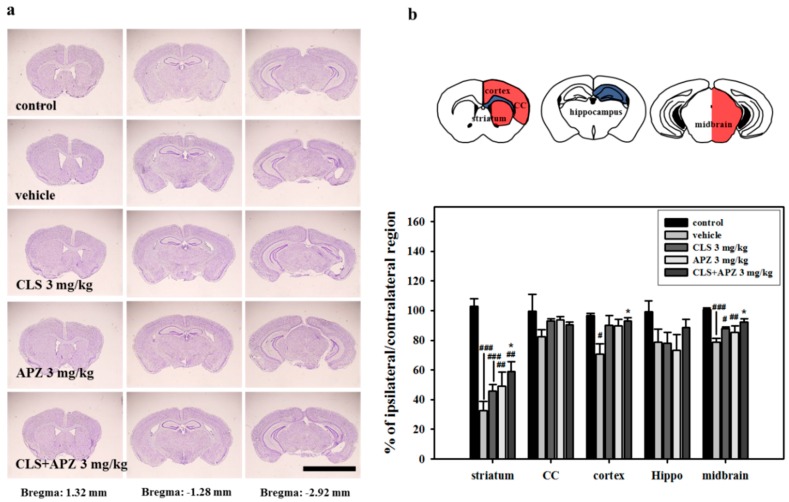
Comparison of ipsilateral/contralateral atrophy volume in each brain region. Photomicrograph (**a**, cresyl violet stain) and its histogram (**b**) for histological analysis (*n* = 5). Schematic diagram (**b**) shows the regions of the striatum, corpus callosum, cortex, hippocampus, and midbrain of the brain. Each region was located at 1.32, −1.28, and −2.92 mm from the bregma. The combined use of APZ and CLS resulted in significantly ameliorated atrophy changes of the ipsilateral striatum, cortex and midbrain compared to vehicle-treated mice. CC, corpus callosum; hippo, hippocampus. ^#^ *p* < 0.05, ^##^ *p* < 0.01 and ^###^ *p* < 0.001 versus control mice; * *p* < 0.05 versus vehicle-treated mice; scale bars = 5 mm.

**Figure 3:**
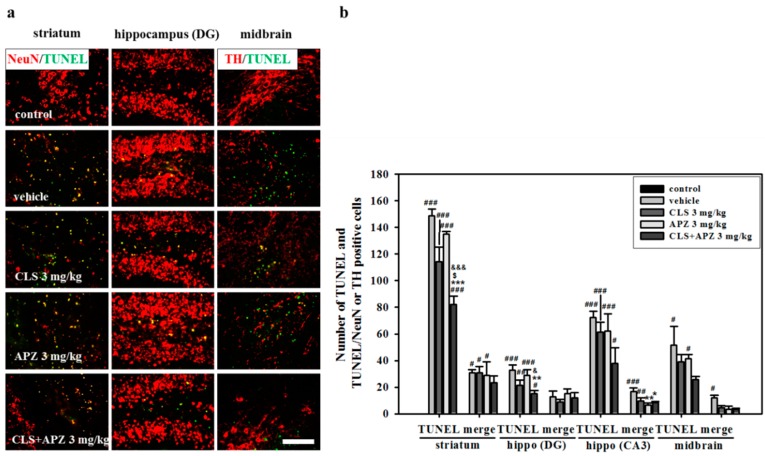
Effect of APZ augmentation on neuronal cell death in CLS-treated mice. Photomicrograph (**a**) and its histogram (**b**) for apoptosis marker TUNEL (terminal deoxynucleotidyl transferase dUTP nick end labeling) and TUNEL/neuronal cell marker NeuN or dopaminergic neuron marker TH in the striatum, hippocampus, and midbrain (*n* = 5). TUNEL positive cells were significantly decreased by the combined use of APZ and CLS in the striatum and DG of the hippocampus. The combined use of APZ and CLS also reduced the number of TUNEL/NeuN-double positive cells in the CA3 of the hippocampus. DG, dentate gyrus; CA3, cornu ammonis; hippo, hippocampus. ^#^ *p* < 0.05, ^##^ *p* < 0.01 and ^###^ *p* < 0.001 versus control mice; * *p* < 0.05, ** *p* < 0.01 and *** *p* < 0.001 versus vehicle-treated mice; ^$^ *p* < 0.05 versus CLS-treated mice; ^&^ *p* < 0.05 and ^&&&^ *p* < 0.001 versus APZ-treated mice. Scale bars = 100 μm.

In the histological analyses of the degree of atrophy, severe atrophic changes in vehicle-treated mouse brain were observed in the striatum, cortex, and midbrain compared to the control brains. The degree of atrophy was particularly high in the striatum, suggesting that this was the primary MCAO lesion site. However, these atrophic changes were countered by treating the mice with APZ and CLS (Figure 2: striatum, F_(4,20)_ = 29.964, *p* < 0.001; cortex, F_(4,20)_ = 5.103, *p* = 0.005; midbrain, F_(4,20)_ = 9.952, *p* < 0.001). In the apoptosis assays, no terminal deoxynucleotidyl transferase-mediated dUTP nick end labeling (TUNEL)-positive cells were detected in the control mice. However, TUNEL-positive cells were significantly increased in all regions examined in the vehicle-treated mice, particularly in the striatum and cornu ammonis 3 (CA3) of the hippocampus. Lower numbers of TUNEL-positive cells were detected in all regions examined in drug-treated mice compared to vehicle-treated mice. However a significant decrease was observed only in the striatum and dentate gyrus (DG) of the hippocampus in mice treated with APZ and CLS (striatum, F_(4,20)_ = 165.454, *p* < 0.001; hippocampus (DG), F_(4,20)_= 9.434, *p* < 0.001). In addition, mice treated with APZ and CLS showed a significantly lower number of TUNEL-positive cells in the striatum compared to mice treated with APZ or CLS alone (striatum, F_(4,20)_ = 165.454, *p* < 0.001). In terms of neuronal cell death, significantly lower numbers of TUNEL/neuronal nuclei (NeuN)-double positive cells were detected in the CA3 of the hippocampus of mice receiving combined treatment compared with vehicle-treated mice (Figure 3). These results suggest that the combined use of APZ and CLS reduced prominent atrophic changes, particularly in the striatum and hippocampus via inhibition of neuronal cell death.

### 2.3. Treatment Effects on Proliferation, Differentiation, and Synaptic Formation in Neuronal Progenitor Cells

**Figure 4:**
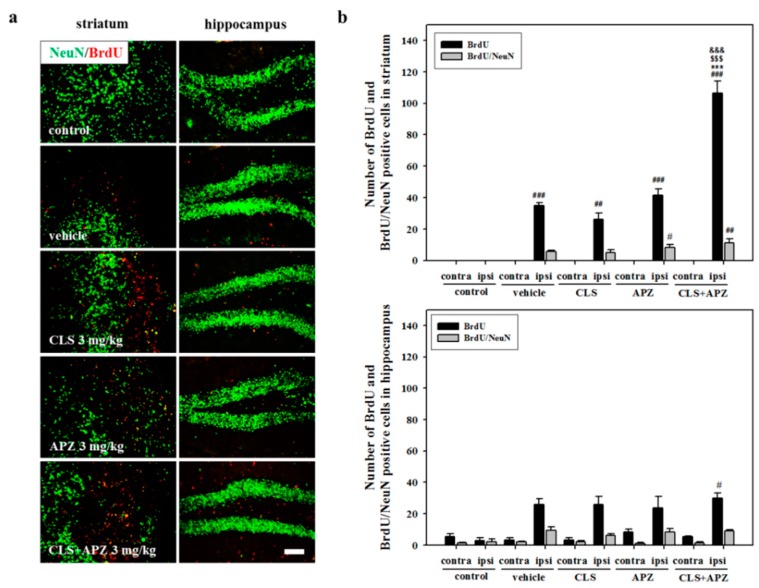
Effect of APZ augmentation on proliferation and differentiation from neuronal progenitor cells in CLS-treated mice. Photomicrograph (**a**) and its histogram (**b**) for 5-bromo-2′-deoxyuridine (BrdU) and BrdU/NeuN-double positive cells in the striatum and hippocampus (*n* = 5). The combined use of APZ and CLS significantly increased the number of BrdU-positive cells in the striatum compared to vehicle-, CLS-, and APZ-treated mice. Contra, contralateral; ipsi, ipsilateral. ^#^ *p* < 0.05, ^##^ *p* < 0.01 and ^###^ *p* < 0.001 versus control mice; *** *p* < 0.001 versus vehicle-treated mice; ^$$$^ *p* < 0.001 versus CLS-treated mice; ^&&&^ *p* < 0.001 versus APZ-treated mice. Scale bars = 100 μm.

**Figure 5:**
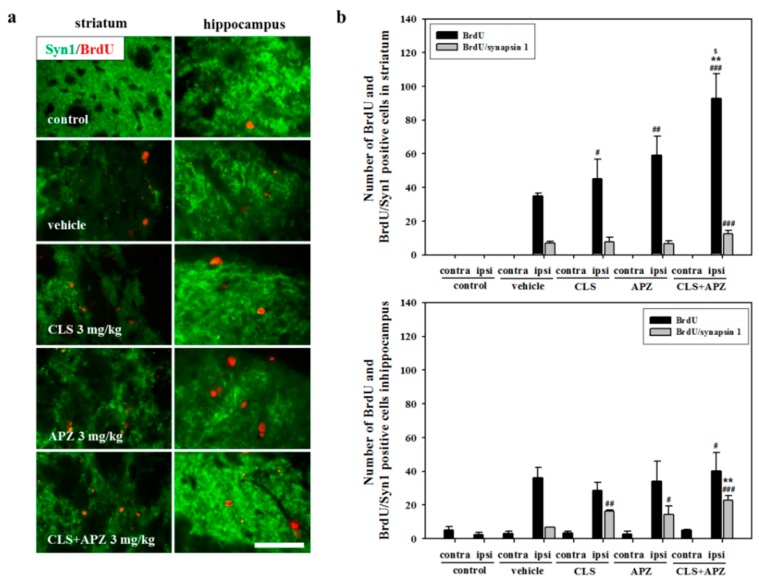
Effect of APZ augmentation on synaptic formation in proliferating progenitor cells in CLS-treated mice. Photomicrograph (**a**) and its histogram (**b**) for BrdU and BrdU/Syn1-double positive cells in the striatum and hippocampus (*n* = 5). The combined use of APZ and CLS significantly increased the number of BrdU/Syn1-double positive cells in the hippocampus compared to the vehicle treatment. Contra, contralateral; ipsi, ipsilateral. ^#^ *p* < 0.05, ^##^ *p* < 0.01 and ^###^ *p* < 0.001 versus control mice; ** *p* < 0.01 versus vehicle-treated mice; ^$^ *p* < 0.05 versus CLS-treated mice. Scale bars = 50 μm.

In the analysis of neuronal progenitor cell proliferation, vehicle-treated mice showed a significant increase in 5-bromo-2′-deoxyuridine (BrdU)-positive cells in the ipsilateral striatum and hippocampus compared to the controls. There was no significant difference observed between the single-drug-treated mice. However, mice treated with APZ and CLS showed significantly higher numbers of BrdU-positive cells in the striatum compared to mice treated with vehicle, APZ, or CLS (F_(4,20)_ = 141.227, *p* < 0.001). In the analysis of neuronal progenitor cell maturation, the numbers of BrdU/NeuN-double positive cells were slightly increased by drug treatment, but not significantly so (Figure 4). In the analysis of synaptic formation, more intense immunoreactivity for synapsin 1 (Syn1) was detected in the drug-treated mice compared to controls. In the assessment of proliferating progenitor cells, the number of BrdU/Syn1-double positive cells was significantly increased by the combined use of APZ and CLS in the striatum (F_(4,20)_ = 6.188, *p* = 0.002) and hippocampus (F_(4,20)_ = 11.332, *p* < 0.001), compared to that in the controls. In addition, the combined use of APZ and CLS significantly increased BrdU/Syn1-double positive cells in the hippocampus, compared to that in the vehicle-treated mice (Figure 5: F_(4,20)_ = 11.332, *p* < 0.001). These results suggest that the combined use of APZ and CLS enhances proliferation, maturation, and synaptic formation in neuronal progenitor cells.

### 2.4. Effect on Phosphorylation of CREB

**Figure 6:**
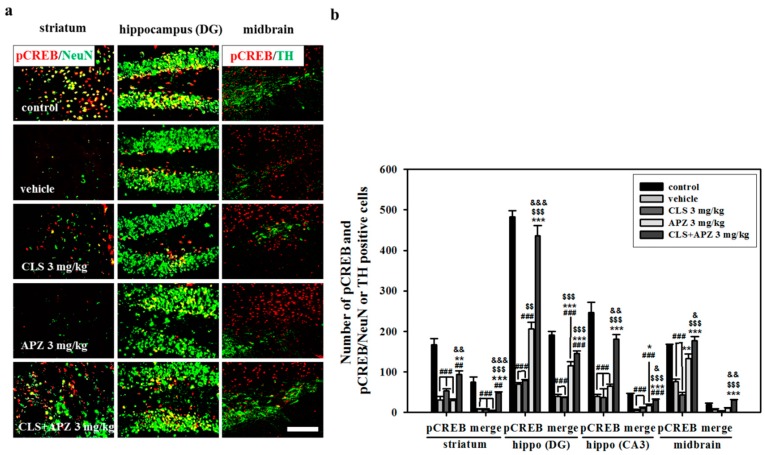
Effect of APZ augmentation on the phosphorylation of cyclic adenosine monophosphate response element binding protein (CREB) and its localization in CLS-treated mice. Photomicrograph (**a**) and its histogram (**b**) for pCREB in all regions examined, pCREB/NeuN in the striatum and hippocampus (DG and CA3) and pCREB/TH in the midbrain. pCREB-positive cells were significantly increased by the combined use of APZ and CLS in all regions examined (*n* = 5). Additionally, pCREB/NeuN or TH-double positive cells were recovered in the striatum, hippocampus and midbrain by the combined use of APZ and CLS. DG, dentate gyrus; CA3, cornu ammonis; hippo, hippocampus. ^##^ *p* < 0.01 and ^###^ *p* < 0.001 versus control mice; * *p* < 0.05, ** *p* < 0.01 and *** *p* < 0.001 versus vehicle-treated mice; ^$$^ *p* < 0.01 and ^$$$^ *p* < 0.001 versus CLS-treated mice; ^&^ *p* < 0.05, ^&&^ *p* < 0.01 and ^&&&^ *p* < 0.001 versus APZ-treated mice. Scale bars = 100 μm.

In all the regions examined, phospho-CREB (pCREB)-positive cells in vehicle-treated mice were significantly decreased compared to those in control mice (Figure 6). Mice treated with APZ and CLS showed significantly higher numbers of pCREB-positive cells in the hippocampus and midbrain compared to mice treated with vehicle, APZ alone, or CLS alone (hippocampus (DG), F_(4,20)_ = 52.737, *p* < 0.001; hippocampus (CA3), F_(4,20)_ = 344.847, *p* < 0.001; midbrain, F_(4,20)_ = 181.128, *p* < 0.001). In the striatum, mice receiving combined treatment showed higher numbers of pCREB-positive cells compared to mice treated with vehicle and APZ alone (striatum, F_(4,20)_ = 80.294, *p* < 0.001). pCREB/NeuN-double positive cells were decreased in all the regions examined in vehicle-treated mice, but these changes were reversed by the combined use of APZ and CLS, particularly in the striatum and hippocampus (striatum, F_(4,20)_ = 52.737, *p* < 0.001; hippocampus (DG), F_(4,20)_ = 103.661, *p* < 0.001; hippocampus (CA3), F_(4,20)_ = 113.703, *p* < 0.001). Similar to pCREB/NeuN-double positive cells, the numbers of pCREB/tyrosine hydroxylase (TH)-double positive cells of vehicle-treated ischemic mice were significantly recovered in the midbrain by the combined use of APZ and CLS (Figure 6: F_(4,20)_ = 44.931, *p* < 0.001). These results suggest that APZ and CLS have a synergistic effect on the activation of CREB signaling.

## 3. Discussion

We have reported here that the combined use of APZ and sub-active doses of CLS resulted in significant recovery from depressive behaviors concomitant with the inhibition of neuronal cell death and the enhancement of proliferation of neuronal progenitor cells following mild chronic stress after an ischemic insult. This demonstrates the potentiation action of the neuroprotection and neurogenesis of APZ. Our data also suggest that the antidepressant effects of APZ may involve a common CREB signaling pathway, shared with CLS; this may be the means by which APZ augments the effects of CLS, leading to the synergistic protection of neuronal cells and the promotion of neurogenesis after ischemia.

APZ, approved for treatment of schizophrenia, is sometimes referred to as a third-generation antipsychotic because it has a unique pharmacological profile [13,26]. It is a partial agonist of serotonin 5-HT_1A_ and 5-HT_7_ receptors, an antagonist of serotonin 5-HT_2A_ and 5-HT_6_ receptors, and a partial agonist of the D_2_ dopamine receptors. Thus, APZ is described as a dopamine-serotonin system stabilizer [12,13]. Adjunctive therapy with low-dose APZ is effective for some complex post-stroke emotional disorders [14]. APZ also improves cognitive function via enhancement of cell proliferation and neuroblast differentiation from neuronal progenitor cells in the hippocampus [27,28]. These clinical and pre-clinical studies demonstrate the usefulness of APZ in the treatment of major depressive disorder through direct effects on neurotransmitter systems [13,29,30]. However, APZ also has a beneficial effect on neuronal regeneration following neuronal loss in the brain after an ischemic assault through indirectly promoting neuronal survival, proliferation, and differentiation from neural progenitor cells [31,32].

CLS is a potent inhibitor of type 3 phosphodiesterase (PDE3) which mainly activates PDE3/cAMP-dependent intracellular signaling by preventing the degradation of cAMP, resulting in increased CREB phosphorylation [17]. Brain-derived neurotrophic factor (BDNF) coupled to the phosphorylation of CREB is a key modulator of CLS action for cerebral ischemic injury and neurogenesis, which contributes to neuronal survival, proliferation, and differentiation from progenitor cells [33,34,35]. CLS inhibits apoptotic cell death in neurodegenerative disease models such as ischemic stroke and chronic cerebral hypoperfusion [23,36], and helps in the secondary prevention of ischemic stroke [18,24]. CLS also activates a CREB-mediated signaling pathway and enhances the proliferation and differentiation of neuronal progenitor cells [16,37].

A potential novel target for post-stroke treatment with regard to functional recovery is the prevention of primary and secondary neuronal injury by ischemic insults. Atypical antipsychotics such as APZ may show better therapeutic efficacy for post-stroke depression if they are combined with a prescription drug for stroke that has a similar or common pathway for neuroprotection and neurogenesis. Because CLS is used clinically as a general prescription drug for the secondary prevention of stroke, we selected this drug to determine whether augmentation with APZ increases the degree of neuroprotection and neurogenesis. In assessments of two different kinds of behavioral tests reflecting mood changes and memory impairments, these effects did not consistently appear, and behavioral results such as anhedonia and despair-like behavior did not appear simultaneously. Despite the limitations of these animal behavioral outcomes, the combined use of chronic APZ with a sub-active dose of CLS resulted in a marked amelioration of depressive behaviors at certain points in the experiments. The combined treatment also prevented atrophic changes, particularly in the striatum and hippocampus, via inhibition of apoptotic cell death and enhancement of neurogenesis, with the two drugs exerting clear and synergistic antidepressant and neuroprotective effects.

In oxidative stress or depression-induced oxidative stress, APZ is highly effective for the prevention of cell death [38,39]. Some researchers have hypothesized that activation of serotonin 5-HT_1A_ or dopamine D_2_ receptors does not underlie the neuroprotectant effects of APZ [31]. Chronic administration of APZ increases protein phosphatase 1 (PP1) activity by inactivating the cAMP/protein kinase A/inhibitor 1 pathway followed by de-phosphorylation of calcium/calmodulin-dependent protein kinase II (CaMKII) by PP1 in the cytoplasm, resulting in nuclear translocation [25]. Nuclear CaMKII functions in transcriptional regulation of the neurotrophin BDNF through the phosphorylation of diverse nuclear proteins, including CREB [25,40,41]. As shown by the increase in pCREB levels by the combined use of APZ and CLS, our data support our hypothesis that this therapy may enhance the neuroprotective effects and neurogenesis via the co-activation of CREB signaling.

**Figure 7:**
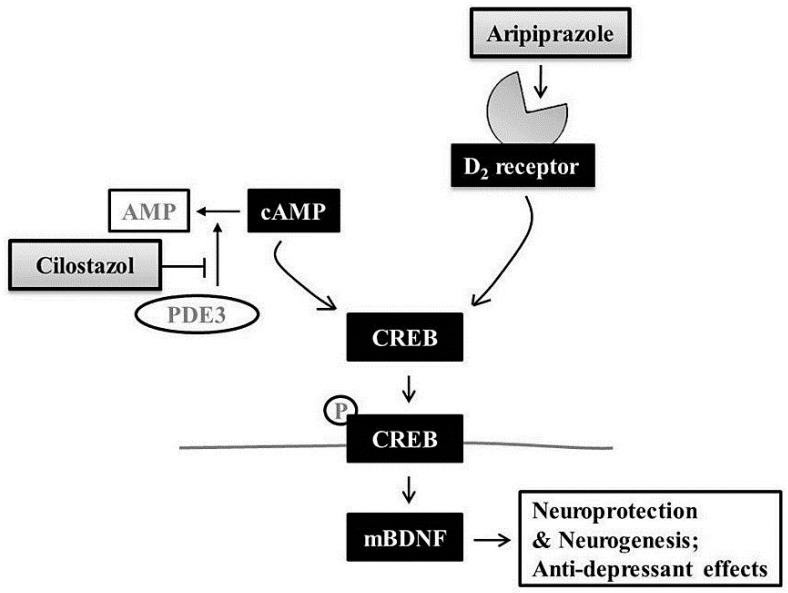
Schematic diagram of the proposed underlying mechanism of APZ augmentation of the antidepressant effects of CLS. PDE3, phosphodiesterase 3; CREB, cyclic adenosine monophosphate response element binding protein; mBDNF, mature brain-derived neurotrophic factor.

Further, PDE3–cAMP intracellular signaling is important for the neuroprotective and neurogenesis effects of CLS, which could promote BDNF expression and a subsequent anti-apoptotic cascade [34,42]. Our previous results suggested that the beneficial effects of CLS involve the activation of CREB/BDNF signaling in post-stroke depression [43]. Therefore, the marked synergistic effect on depression-like behaviors and neuronal survival by the combined use of APZ and CLS may be associated with the convergent activation of CREB/BDNF signaling pathways by APZ and CLS [25,44] (Figure 7). However, we cannot exclude the possibility that dopamine D_2_ receptors may also play a major role in APZ’s mechanism of action, nor can we exclude more complex mechanisms such as agonistic activity of 5HT_1A_ receptors and antagonistic activity of 5-HT_2A_ receptors. The exact mechanisms underlying the action of neuroprotection and neurogenesis of APZ are still unclear. Therefore, whether the anti-depressive effects in our study arose from the neuroprotective effects of APZ, or from the cognitive improvement via neurogenesis after APZ treatment is unclear and needs to be elucidated through further study.

## 4. Materials and Methods

### 4.1. Animals

C57BL/6 male mice (10 weeks of age) were purchased from Dooyeol Biotech (Seoul, Korea). The mice were housed at 22 °C under alternating 12 h cycles of dark and light and allowed tap water ad libitum with a commercial diet throughout the study. All experiments were approved by the Pusan National University Animal Care and Use Committee in accordance with the recommendations of the Guide for the Care and Use of Laboratory Animals of the National Institutes of Health (approval number PNU-2015-0850; 21 April 2015). The mice were randomly divided into five groups (control (non-treated normal mice), vehicle (non-drug-treated mice undergoing the CMS process), CLS, APZ, and CLS + APZ). Each group consisted of six mice.

### 4.2. Focal Cerebral Ischemia

To induce focal cerebral ischemia, occlusion of the middle cerebral artery (MCA) using the intraluminal filament technique was employed. A fiber-optic probe was affixed to the skull over the MCA to measure regional cerebral blood flow using a PeriFlux Laser Doppler System 5000 (Perimed, Stockholm, Sweden). A left MCAO model was produced using a silicon-coated 7-0 monofilament, which was advanced through the internal carotid artery to occlude the MCA. The filament was withdrawn 30 min after MCA occlusion, and reperfusion was confirmed using laser Doppler. After surgery, we isolated these mice from the controls and monitored their condition daily for signs of decreased activity and decline in food intake. We did not observe any signs of these conditions. All operations were performed with the administration of 2.0% isoflurane (VSP Corporation, Hanami, Korea) anesthesia to minimize mouse suffering, using a model VIP 3000 calibrated vaporizer (Midmark, Orchard Park, OH, USA).

### 4.3. Chronic Mild Stress

The CMS procedure was applied as previously described, with slight modifications [45]. The CMS regimen included seven different stressors as follows: food and water deprivation for 20 h, water deprivation for 18 h, 45° cage tilt for 17 h, overnight illumination for 36 h, soiled cage for 21 h, swimming in 4 °C water for 5 min, and paired caging for 2 h. CMS treatments were administered on a schedule, day and night, for 16 consecutive days, starting from 2 days post-MCAO.

### 4.4. Drug Administration

APZ (7-{4-[4-(2,3-Dichlorophenyl)piperazin-1-yl]butoxy}-3,4-dihydroquinolin-2(1*H*)-one) and CLS {6-[4-(1-cyclohexyl-1*H*-tetrazol-5-yl) butoxy]-3,4-dihydro-2-(1*H*)-quinolinone} were donated by Otsuka Pharmaceutical (Tokushima, Japan). The APZ and CLS were administered orally from 2 days after MCAO for 16 successive days. Drugs were used at a concentration of 3 mg/kg body weight. The APZ and CLS were dissolved in 10% dimethyl sulfoxide (DMSO) and in distilled water, respectively.

### 4.5. Bromodeoxyuridine Labeling

For labeling of proliferating cells, a synthetic thymidine analog, BrdU (Sigma-Aldrich Corporation, St. Louis, MO, USA) was employed, which becomes incorporated into a dividing cell’s DNA during the S-phase of the cell cycle. All animals were injected with BrdU (50 mg/kg intraperitoneally) for 5 successive days after MCAO.

### 4.6. Behavioral Experiments

Behavioral experiments such as the open field, sucrose preference, and forced swim test were performed once a week from the 4th week to the 6th week after MCAO. Morris water maze tests were performed at the 6th week after MCAO daily for a successive 4-day period. All behavioral experiments were performed at 22 ± 1 °C. The behavioral experiments were performed as previously described, with slight modifications [46,47].

#### 4.6.1. Open Field Test

The open field test was employed to measure anxiety-like behavior. Following adaptation for 10 min in a white box (30 × 30 × 40 cm^3^), the total distance and number of entries into the center area were measured for 30 min by using the SMART 2.5.18 video tracking system (Panlab, S.L.U., Barcelona, Spain) in a quiet room.

#### 4.6.2. Sucrose Preference Test

A sucrose preference test was employed to measure anhedonia. Before MCAO, all mice were habituated to 1% sucrose solution (AMRESCO Inc., Solon, OH, USA) for 24 h. For the experiment, the mice were deprived of food and water for 20 h, after which both 1% sucrose solution and water bottles were placed in the cage for 1 h. The consumption of sucrose solution and water was determined by measuring the weight of these bottles.

#### 4.6.3. Forced Swim Test

A forced swim test was employed to measure despair-like behavior. Mice were exposed to 25 °C water for 15 min in a glass cylinder (15 cm in height × 10 cm diameter) one day before the test. Their behavior was recorded using a digital camera (E8400, Nikon Corporation, Tokyo, Japan) for 5 min. We also recorded the time from when the mouse entered the cylinder until it stopped struggling.

#### 4.6.4. Morris Water Maze Test

The Morris water maze test was employed to measure spatial memory. A tank (100-cm diameter and 50-cm height) was employed, and a platform was placed 0.5 cm beneath the surface of the water. Before MCAO, the mice were trained for 4 successive days to go to the target. The experiment consisted of 5 trials per day for 4 successive days, and each trial lasted for 90 s or until the mouse climbed onto the platform. The time taken and distance travelled to reach the platform were recorded using the SMART 2.5.18 video tracking system (Panlab, S.L.U.).

### 4.7. Histological Assessment

Intraperitoneal perfusion of the mice with saline followed by 4% paraformaldehyde in phosphate buffered saline (PBS) was performed under anesthesia with 8% chloral hydrate. After removal of the brains, tissues were fixed again in the same fixative for 24 h at 4 °C, and immersed in a 30% sucrose solution for 72 h at 4 °C. To evaluate brain damage or atrophy, frozen 30-μm-thick sections were stained with 0.1% cresyl violet (Sigma-Aldrich Corporation, St. Louis, MO, USA). The contralateral and ipsilateral subarea sizes of each section including the striatum, corpus callosum, cortex, hippocampus, and midbrain were measured using i-solution (IMT i-Solution Inc., Burnaby, BC, Canada). Atrophy volume was expressed as a percentage: atrophy volume = subarea volume of the ipsilateral hemisphere/subarea volume of contralateral hemisphere × 100.

### 4.8. Terminal Deoxynucleotidyl Transferase-Mediated dUTP Nick End Labeling Assay

For characterization of apoptotic neuronal death, TUNEL was performed using a DeadEnd™ Fluorometric TUNEL System kit (Promega Corporation, Madison, WI, USA), according to the manufacturer’s instructions. TUNEL-positive cells were counted using a fluorescence microscope (Carl Zeiss, Inc., Gottingen, Germany), and quantitative analysis was performed blindly. Data are presented as the total number of apoptotic cells.

### 4.9. Immunofluorescence

Frozen sections (30-μm thickness) were incubated with a blocking buffer (1× PBS/5% normal goat serum/0.3% Triton X-100) for 1 h. Sections were incubated with primary antibodies overnight in an antibody dilution buffer (1× PBS/1% BSA/0.3% Triton X-100) at 4 °C. The antibodies were as follows: BrdU (Cat. OBT0030G, AbD Serotec, Oxford, UK), NeuN (Cat. MAB377; ABN78, Millipore Corporation, Billerica, MA, USA), Syn1 (Cat. ab64581, Abcam, Cambridge, UK), TH (Cat. AB152, Millipore Corporation), and pCREB (Cat. sc-7978-R, Santa Cruz Biotechnology, Santa Cruz, CA, USA). The sections were incubated with a fluorescent secondary antibody (Vector Laboratories, Inc., Burlingame, CA, USA) for 2 h and 4′,6-diamidino-2-phenylindole (DAPI, Invitrogen Corporation, Carlsbad, CA, USA) for 30 min in the dark, respectively. After slide mounting with a mounting medium (Vector Laboratories, Inc.), images were captured using a fluorescence microscope (Carl Zeiss, Inc.).

### 4.10. Data Analyses

Data were analyzed using the Sigmaplot statistical program version 11.2 (Systat Software, San Jose, CA, USA). All data are expressed as mean ± standard error of the mean (SEM). Statistical analysis of the data was performed using one-way or one-way repeated measures analysis of variance (ANOVA) via Tukey’s post hoc comparison when comparing more than two groups. A *p* < 0.05 was considered statistically significant.

## 5. Conclusions

In summary, our current results showed that a lower active dose of APZ with a sub-active dose of CLS could synergistically enhance the antidepressant effects of either drug with particular improvements in the atrophic changes of the primary neurodegenerative sites in the striatum and hippocampus following ischemic assaults. Further attempts to establish a mechanism of action for APZ might focus on our evidence showing an antidepressant effect through neuroprotection and neurogenesis. Collectively, the beneficial effects of APZ in post-stroke depression may be due in part to neuroprotective and neurogenesis properties via a common signaling pathway of CLS, such as the cAMP/CREB signaling pathway, with the primary beneficial actions of the drugs derived from improved monoamine function.

## Abbreviations

ANOVA	analysis of variance
APZ	aripiprazole
BDNF	brain-derived neurotrophic factor
BrdU	5-bromo-2′-deoxyuridine
CA	cornu ammonis
CaMKII	calcium/calmodulin-dependent protein kinase II
cAMP	cyclic adenosine monophosphate
CLS	cilostazol
CMS	chronic mild stress
CREB	cAMP response element binding protein
DAPI	4′,6-diamidino-2-phenylindole
DG	dentate gyrus
MCA	middle cerebral artery
MCAO	middle cerebral artery occlusion
NeuN	neuronal nuclei
PBS	phosphate buffered saline
PDE3	type 3 phosphodiesterase
PP1	protein phosphatase 1
Syn1	synapsin 1
TH	tyrosine hydroxylase
TUNEL	terminal deoxynucleotidyl transferase-mediated dUTP nick end labeling
